# The endocannabinoid system promotes hepatocyte progenitor cell proliferation and maturation by modulating cellular energetics

**DOI:** 10.1038/s41420-023-01400-6

**Published:** 2023-03-25

**Authors:** Bani Mukhopadhyay, Kellie Holovac, Kornel Schuebel, Partha Mukhopadhyay, Resat Cinar, Sindhu Iyer, Cheryl Marietta, David Goldman, George Kunos

**Affiliations:** 1 Laboratory of Physiologic Studies, National Institute on Alcohol Abuse and Alcoholism, National Institutes of Health, Bethesda, MD USA; 2 Laboratory of Neurogenetics, National Institute on Alcohol Abuse and Alcoholism, National Institutes of Health, Bethesda, MD USA; 3 Laboratory of Cardiovascular Physiology and Tissue Injury, National Institute on Alcohol Abuse and Alcoholism, National Institutes of Health, Bethesda, MD USA

**Keywords:** Non-alcoholic fatty liver disease, Transcriptomics, TOR signalling

## Abstract

The proliferation and differentiation of hepatic progenitor cells (HPCs) drive the homeostatic renewal of the liver under diverse conditions. Liver regeneration is associated with an increase in Axin2^+^Cnr1^+^ HPCs, along with a marked increase in the levels of the endocannabinoid anandamide (AEA). But the molecular mechanism linking AEA signaling to HPC proliferation and/or differentiation has not been explored. Here, we show that in vitro exposure of HPCs to AEA triggers both cell cycling and differentiation along with increased expression of *Cnr1, Krt19*, and *Axin2*. Mechanistically, we found that AEA promotes the nuclear localization of the transcription factor β-catenin, with subsequent induction of its downstream targets. Systemic analyses of cells after CRISPR-mediated knockout of the β-catenin-regulated transcriptome revealed that AEA modulates β-catenin-dependent cell cycling and differentiation, as well as interleukin pathways. Further, we found that AEA promotes OXPHOS in HPCs when amino acids and glucose are readily available as substrates, but AEA inhibits it when the cells rely primarily on fatty acid oxidation. Thus, the endocannabinoid system promotes hepatocyte renewal and maturation by stimulating the proliferation of Axin2^+^Cnr1^+^ HPCs via the β-catenin pathways while modulating the metabolic activity of their precursor cells.

## Introduction

Understanding the cellular and molecular mechanisms underlying liver regeneration is of immense interest as such insight could potentially be leveraged to provide effective therapies for life-threatening liver failure, including those caused by alcohol or non-alcoholic liver cirrhosis, hepatocellular carcinoma, viral hepatitis, toxin-induced liver damage or other forms of fulminant hepatic failure or chronic liver disease [1, 2]. Such pathology is a largely unmet clinical need, and, according to a report from the Centers for Disease Control and Prevention, in 2017 there were 4.5 million Americans diagnosed with chronic liver diseases resulting in 41,743 deaths. One known molecular pathway in liver metabolism and disease is the endocannabinoid-CB_1_ receptor (CB_1_R, which is encoded by *Cnr1*) system (ECS) [3–6]. Further, this pathway plays a critical role in liver regeneration [7, 8] and also modulates liver function in alcohol-induced steatosis, non-alcoholic fatty liver disease, and hepatocellular carcinoma [9–11].

Oval cells, which are liver cells with a high nuclear:cytoplasmic ratio and an ovoid nucleus, are generally considered hepatic progenitor cells (HPCs) [12]. They are generated from biliary epithelial cells and differentiate into hepatocytes [13]. However, significant variability in their morphology and gene expression profiles suggests that ovoid cells are a heterogeneous population, and some may be more likely than others to differentiate into distinct cell types [14]. Their exact role in liver regeneration is still debatable, but their presence in liver development is very well established [15]. Recently, liver cells expressing LGR5 or Axin2 have been proposed as unique precursors of hepatocytes [16, 17]. Self-renewing Axin2^+^ hepatic progenitor cells are the source of clones of hepatocytes expanding from the central vein towards the portal vein and are responsible for the natural homeostatic renewal of the liver [17]. Axin2 expression is regulated by the transcription factor β-catenin, which, in turn, is under the control of Wnt proteins expressed in the stem cell niche [18]. Recently, multiple single-cell RNA-sequencing analyses revealed the existence of various subsets of immature/mature hepatocytes in the mammalian liver, each with a unique metabolic role [19, 20]. The aim of this study was to characterize and explore the role of Axin2^+^Cnr1^+^ HPC cells in liver regeneration and the interplay of the ECS with the β-catenin pathways as a regulatory mechanism. We also investigated the role of the endogenous endocannabinoid, anandamide (AEA), in mitochondrial energy metabolism—in Axin2^+^Cnr1^+^ HPCs at the molecular level during liver regeneration.

## Results

### Partial hepatectomy induces *Axin2* and *Cnr1* expression in mouse liver

**Fig. 1:**
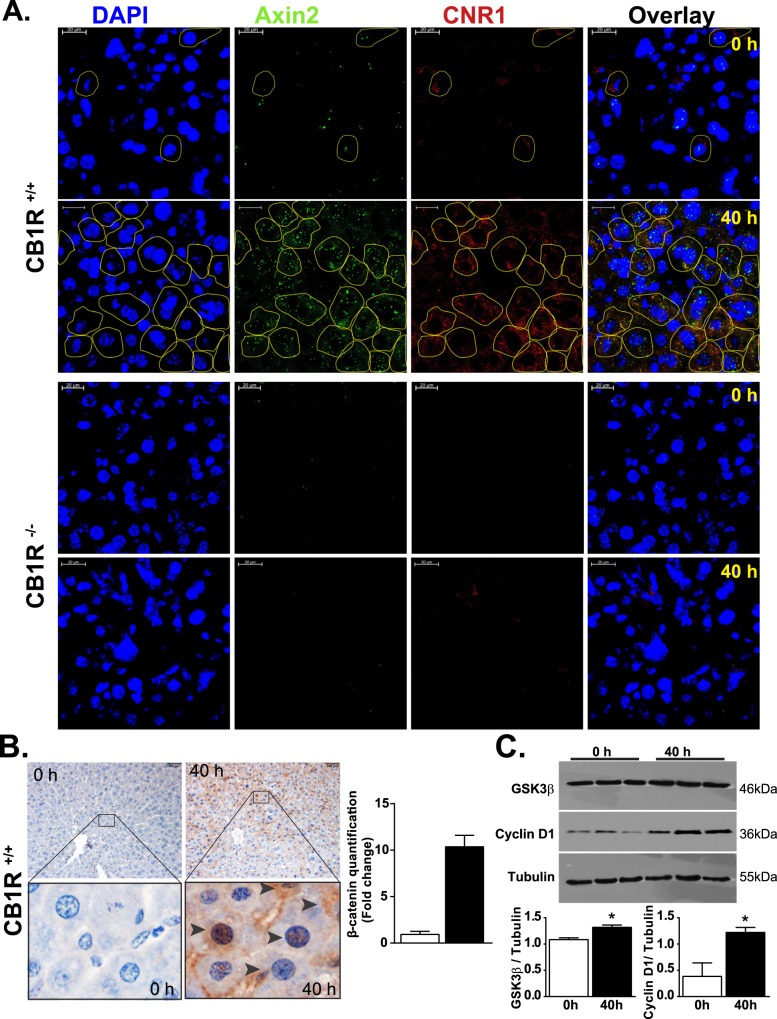
Expression of *Axin2* and *Cnr1* in post-hepatectomy remnant liver from wild-type (CB_1_R^+/+^) and CB_1_R^−/−^ mice. **A** Representative RNAScope analyses of *Axin2*, *Cnr1* and their co-expression in wild-type (CB_1_R^+/+^) and CB_1_R^−/−^ liver samples at 0 and 40 h after PHx. **B** Representative immunohistochemical analyses and quantification of β-catenin protein expression in 0 h control and 40 h post-PHx liver from wild-type mice. **C** Representative western blot analyses and quantification by densitometry of the β-catenin target proteins GSK3β and Cyclin D1 in 0 h control and 40 h post-PHx liver samples.

To explore the potential interaction between self-renewing Axin2^+^ cells and the ECS and its role in liver regeneration, we used the in situ hybridization RNAscope technique to analyze the mRNA levels of *Axin2* and *Cnr1* in the remnant liver at 0 and 40 h following 2/3rd partial hepatectomy (PHx) in mice. We found a strikingly greater degree of *Axin2* expression at 40 h post-PHx compared to baseline, which was accompanied by greater expression of *Cnr1*, and there was notable co-localization of *Cnr1* and *Axin2* staining in the same cells (Fig. 1A). These changes paralleled the earlier reported upregulation of AEA production in the regenerating liver [7]. The protein expression of β-catenin, the transcriptional regulator of *Axin2*, was also notably greater at 40 h post-PHx compared to baseline (Fig. 1B). Two other targets of β-catenin, GSK3β and the cell cycle marker Cyclin D1, were significantly induced at 40 h relative to 0 h post-PHx (Fig. 1C).

### Activation of CB_1_R induces the proliferation of mouse and rat hepatocyte progenitor cells

**Fig. 2:**
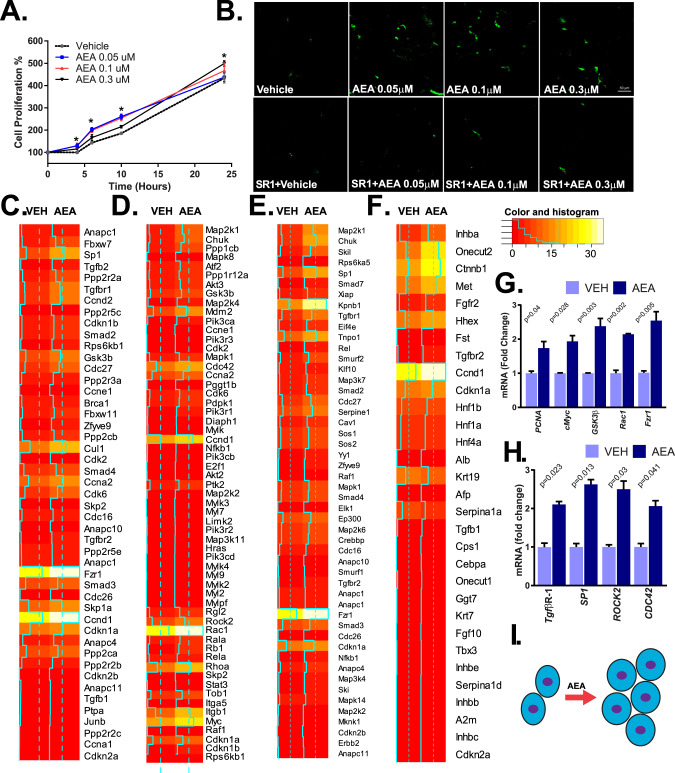
Effect of AEA on the proliferation and transcriptome analyses reveal distinct modulation of cell cycle and cell differentiation pathways by AEA in mouse HPCs. **A** A concentration-dependent increase in proliferation of cells exposed to 50–300 nM anandamide. **B** Inhibition of the pro-proliferative effect of AEA by the CB_1_R antagonist SR1 (rimonabant), as monitored by BrdU incorporation and detected by fluorescence confocal microscopy in BMOL. **C**–**F** RNA-seq analyses of six pooled samples of BMOL cells treated with vehicle (VEH) or AEA (300 nM) are presented as heat maps. These heatmap data reveal AEA-induced genes involved in cell cycle regulation, including regulation of G1/S transition (**C**) and Ras and Rho proteins (**D**); and cellular differentiation, including TGFß-receptor signaling (**E**) and transcription factors involved in hepatocyte lineage segregation (**F**). Differentially expressed genes are highlighted by color coding and by a solid blue line in the heatmap. **G** Real-time PCR validation of five highly regulated target genes in cell cycle pathways (PCNA, cMyc, GSK3β, Rac1, and Fzr1) from three additional samples (*n* = 3/group, * *P* < 0.05 AEA-treated vs vehicle-treated cells). **H** Real-time PCR validation of four target genes in cell differentiation pathways (TGFβR-1, SP1, ROCK2, and CDC42) were verified by real-time PCR analyses (*n* = 3/groups, *P < 0.05 AEA-treated vs vehicle-treated cells). **I** Schematic summary model of the effect of AEA in HPC.

Next, we wanted to test the functional consequences of AEA signaling on HPC proliferation. Thus, we synchronized the mouse hepatocyte progenitor cell line BMOL and the rat progenitor cell line LE2 by serum deprivation, and we monitored cell proliferation for 24 h in the presence of vehicle or different concentrations of AEA. Cell proliferation was monitored by the reduction of tetrazolium salt by cellular dehydrogenases and by BrdU labeling, followed by spectrometry and confocal microscopy. We found enhanced proliferation in the presence of 50 nM AEA compared to vehicle, but less so at higher AEA concentrations (Fig. 2A, B and Supplementary Fig. S1A, B). However, cell proliferation slightly slowed at 24 h in the presence of 50 nM AEA compared to 0.3 µM AEA, possibly due to AEA’s instability in the media. Therefore, we used 0.3 µM AEA throughout the study for the 24-h endpoint. The effects of AEA were abrogated in the presence of the CB_1_R antagonist rimonabant (SR1) (Fig. 2B and Supplementary Fig. S1B), indicating that they are mediated by CB_1_R.

### AEA modulates cell cycle and cellular differentiation pathways in mouse progenitor cells

To understand the mechanistic role of AEA in cell proliferation of HPCs, we performed transcriptome analyses of mouse HPCs (BMOL) using a pool of six samples from such cells treated either with vehicle or AEA at 0.3 µM. GeneGo analyses of the transcriptome indicated that AEA markedly upregulated the expression of cell cycle proteins involved in G1/S transition, as well as Ras and Rho proteins in HPCs. Transcripts differentially expressed by >20% are illustrated in a heatmap (Fig. 2C, D).

The most striking effects of AEA on gene expression were on components of cellular differentiation pathways (Fig. 2EF). The most robustly induced pathway was the TGFβ receptor signaling pathway, of which 56 genes were altered in response to AEA treatment. Similarly, large differences in transcript abundance were observed in a gene network laden with transcription factors involved in the segregation of the hepatocytic lineage. The AEA-induced enrichment of genes within these two pathways was statistically significant. The top inducible targets were *Ccnd1* (encoding cyclin D1) and its regulator *Ctnnb1* (encoding β-catenin) (Fig. 2C). Induction of key target genes (*TgfβR-1*, *Sp1*, *Rock2*, and *Cdc42*) was verified by real-time PCR (Fig. 2G). AEA-induced upregulation of five genes, *Pcna*, *Myc*, *Rac1*, *Gsk3β*, and *Fzr1*, was confirmed by real-time PCR, with the degree of upregulation being consistent with the transcriptome data (Fig. 2H).

**Fig. 3:**
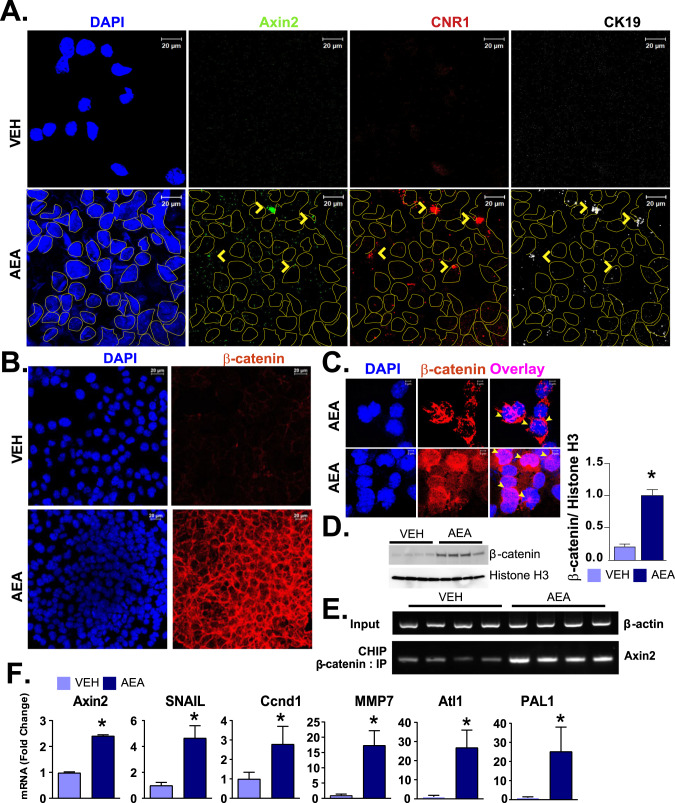
AEA-induced co-expression of Axin2, CB_1_R, and CK19 (*Krt19*) mRNA, as well as that for β-catenin and its target genes, in mouse HPCs. **A** RNAscope analyses of the effect of AEA (300 nM) on the expression of *Axin2, Cnr1*, and *CK19* and in BMOL. Yellow arrows in panel A illustrate co-expression of the three mRNAs in the same cell. Co-expression is also illustrated by an overlay. **B**, **C** Effect of AEA (300 nM) on β-catenin protein localization in the nuclear membrane/nucleus in BMOL cells, as visualized by confocal immuno-histochemistry, including two sets of overlaid images at higher magnifications for AEA group. **D** Immunoblot analyses of β-catenin from nuclear fraction obtained four independent experiments and their quantifications. Histone H3 was used as a nuclear fraction validation marker. **E** CHIP analyses of Axin-2 promoter pulled by β-catenin antibody. Input DNA control was validated by β-actin. **F** Real-time PCR analysis of AEA induction of β-catenin target genes *Axin2, Mmp7, Snail, Ccnd1*, *Atl1*, and *PAI1* (*n* = 3/groups, *P < 0.05 compared to vehicle-treated cells.

Both *Ccnd1* and *Axin2* were induced in mouse liver by PHx, and both are known to be regulated by β-catenin [21, 22]. To further explore the relationship among *Axin2*, *Cnr1*, and an additional progenitor cell marker *Krt19* (encoding CK19) in HPCs, we performed RNAscope analyses in both mice and rat HPCs. HPC proliferation induced by 0.3 µM AEA was associated with increased expression of *Axin2*, *Cnr1*, and *Krt19*, and there was a partial co-expression of these three transcripts in the same cells (Fig. 3A).

### AEA induces nuclear localization of β-catenin in mouse and rat HPCs

**Fig. 4:**
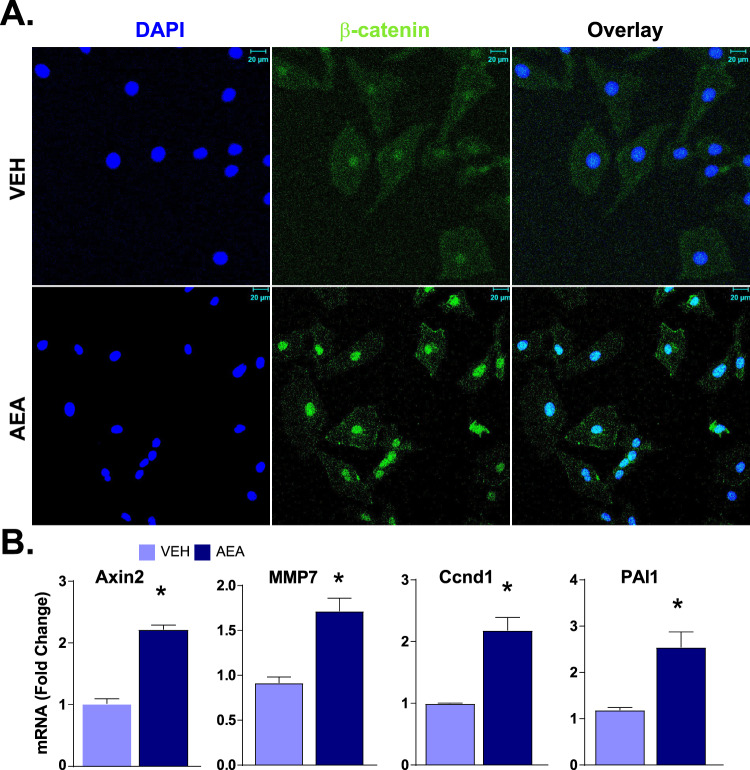
AEA induces the expression of β-catenin and its target genes in rat HPCs. **A** Fluorescence immune-histochemistry for β-catenin (middle), as well as DAPI staining (left), of LE2 cells treated with AEA (300 nM) and visualized by confocal microscopy. Co-localization is shown by overlay (right). **B** Real-time PCR analyses of the expression of β-catenin target genes Axin2, Mmp7, Ccnd1, and PAL1 in the same cells (*n* = 3/groups, *P < 0.05 compared to vehicle).

The Wnt-β-catenin pathway in self-renewing Axin2^+^ cells plays a critical role in hepatocyte homeostasis in the liver [17]. Exposure of rat HPCs to 0.3 µM AEA triggered an increase in the gene and protein expression of β-catenin and induced its translocation to the nucleus (Fig. 4A). Similar changes were observed in mouse HPCs with prominent localization of β-catenin to the nuclear envelope (Fig. 3B). At higher magnification, we also observed β-catenin distribution in both nucleus and its envelope after AEA treatment (Fig. 3C). Isolation of nuclear fraction followed by western blot and its quantification from four experiments demonstrated an increase in β-catenin/histone H3 ratio (Fig. 3D). β-catenin is known to regulate Axin2 through its T-cell factor binding site at the promoter [18, 23]. Therefore, we tested with CHIP assay whether it regulate in BMOL cells. We have observed increase in β-catenin binding on *Axin2* promoter upon treatment with AEA in BMOL cells (Fig. 3E). Increase in nuclear localization of β-catenin was associated with the activation of its target genes, including *Axin2*, *Snail*, *Ccnd1*, *Mmp7*, *Atl11* and *Pal1* as documented by real-time PCR in mouse BMOL cells (Fig. 3F), and *Axin2*, *Mmp7*, *Ccnd1*, and *Pal1* in rat LE2 cells (Fig. 4B).

### CRISPR-mediated knockout of β-catenin alters AEA-induced cell proliferation and differentiation in mouse HPCs

**Fig. 5:**
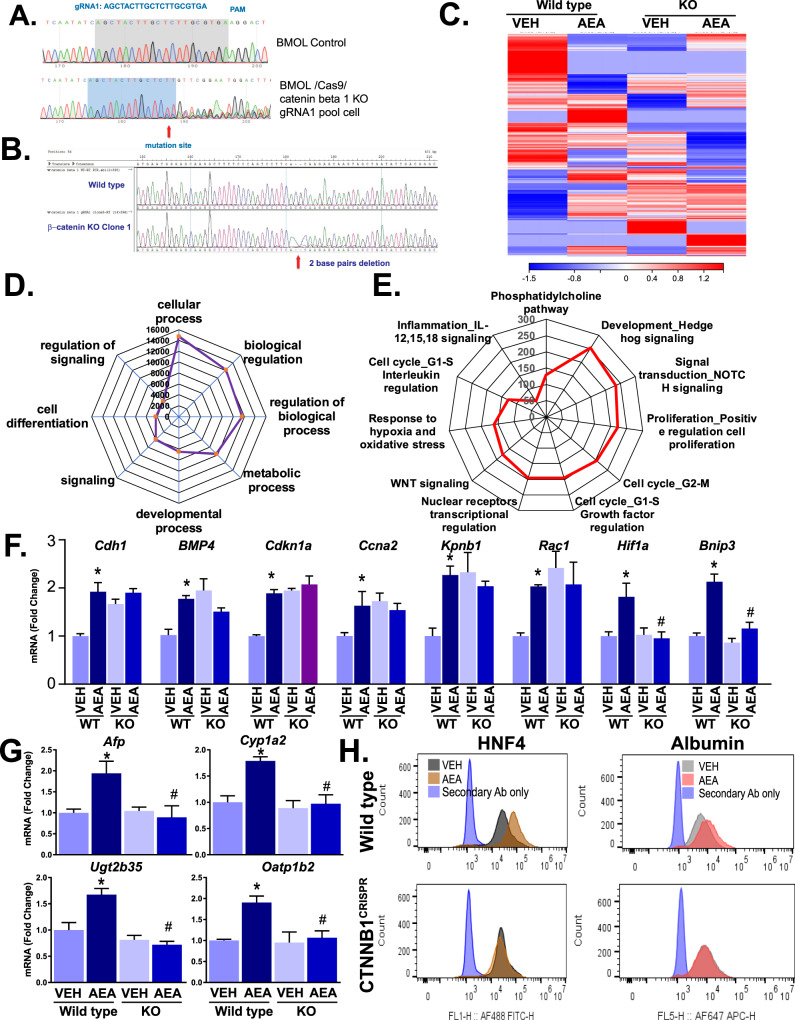
AEA induction of cellular differentiation in mouse HPCs is β-catenin-dependent. **A** Sequence of gRNA for targeting β-catenin in BMOL cells and sequencing of the pooled CRISPER library. **B** Characterization of β-catenin KO clone in BMOL cells by sequencing. **C** Gene expression analyses by RNA-seq, presented as a heatmap, in pooled samples of wild-type and Ctnb1^CRISPR^ (KO) cells treated with vehicle (VEH) or AEA. **D** Radar plot of cellular processes significantly affected by AEA from transcriptome analyses of wild-type and β-catenin KO HPCs. **E** Radar plot of AEA-induced (>1.5-fold increase) genes in wild-type HPCs, which were not similarly induced by AEA in KO cells. **F** Verification of the induction of selected genes from the AEA-induced pool by real-time PCR (*n* = 3/groups, *P* < 0.05 compared to vehicle (VEH) in wild-type (*) or KO cells (#); **G** Real-time PCR analyses of genes involved in hepatocyte maturation (*Afp, Cyp1a2, Ugt2b35* and *Oatp1b2*) in wild-type and β-catenin KO BMOL cells (*n* = 3/groups, *P* < 0.05 compared to vehicle in wild-type (*) or β-catenin KO BMOL cells (#)). **H** Flow cytometry analyses of intracellular HNF4 and albumin in wild-type and Ctnnb1^CRISPR^ BMOL cells treated with vehicle or AEA for 5 days: decrease in hepatocyte-like cell marker HNF4 and albumin in Ctnnb1^CRISPR^ cells.

We next analyzed the functional role of β-catenin in AEA-induced proliferation and differentiation of the mouse HPC BMOL cell line by knocking out *Ctnnb1* via CRISPR technology. We generated β-catenin knockout cells (Ctnnb1^CRISPR^), including a clonal population, which was further confirmed by DNA sequencing (Fig. 5A). A pure, single clonal population was isolated by growing the knockout cells in the presence of puromycin and blasticidin, and a 2-base pair deletion in *Ctnnb1* in the clone was verified by sequencing (Fig. 5B).

Heatmap representation of hierarchical clustering analyses of transcriptome data from vehicle-treated or AEA-treated Ctnnb1^CRISPR^ HPCs demonstrated significant differential gene expression compared to wild-type cells (Fig. 5C). When transcripts were analyzed by the function of the encoded proteins, the list comprised mainly enzymes (Z-score 18.49), transcription factors (11.49), receptors (11), kinases (9.2), proteases (6.2), and phosphatases (data not shown). We further analyzed genes representing markedly enriched pathways that displayed 1.5-fold or greater induction by AEA in pooled samples of wild-type cells and blunted expression of those genes in cells with β-catenin deletion. We detected 4068 such genes and analyzed the molecular processes likely affected by them, using GeneGo. Among significantly affected processes were development (253 genes, including Hedgehog signaling), signal transduction (235 genes including NOTCH signaling), cell proliferation (221 genes in positive regulation), cell cycle (206 genes in G2-M; 195 genes in G1-S, growth factor regulation), development (195 genes in nuclear receptors, transcriptional regulation and 177 genes in WNT signaling), response to hypoxia and oxidative stress (161 genes), interleukin regulation in hepatocytes (128 genes in G1-S interleukin regulation and 59 genes in inflammation involving IL12, IL15, and IL18 signaling) and metabolic pathways (129 genes in phosphatidylcholine pathways) (Fig. 5D, E). These changes in network processes suggested an important role for AEA in β-catenin-mediated regulation of cell proliferation, cell cycle, and differentiation.

Significantly affected networks of pathways involved cMyc, FAK1, GSK3 beta, Paxillin, and STAT3, with a highly significant *P* value (1.90e-48), high g-score (33.12) and high Z-score (20.62Pathways significantly affected by AEA in a β-catenin-dependent manner, as revealed by RNA-seq analyses, were associated with the development and cell cycle (Supplementary Figs. S2–S4). First, WNT-β-catenin signaling in the nucleus of HPCs is part of the cellular differentiation process. Multiple stimuli lead to the induction of β-catenin and its translocation to the nucleus. CB_1_R activation phosphorylates and thus inhibits GSK3β activity [24], which could contribute to the activation of β-catenin. Similar pathways also modulate p38 MAPK/MEK1/ERK1, triggering the phosphorylation of MEF2, which associates with β-catenin and promotes its nuclear retention [25]. Based on Metcore-Clavariate transcriptome analyses, this pathway is significantly affected by AEA (*P* = 1.337e-^4^) with a low false discovery rate (FDR = 7.905e^−4^) (Supplementary Fig S2). AEA also appears to be involved in regulating TGF-β receptor signaling (Supplementary Fig. S3). Interactions of CB_1_R and TGF-β have been reported in cardiac, renal, brain, and liver tissue under various pathophysiological conditions [26–30]. This pathway is highly statistically significant (*P* value = 6.664e^−9^) with a low false discovery rate (FDR = 6.010e^−7^) (Supplementary Fig. S3). Another significantly affected pathway mediates the effects of endocannabinoids on the cell cycle process via regulation of G1 to S transition involving TGF-β (Supplementary Fig. S4). TGF-β factors induce an association of its receptor with the regulatory subunit of protein phosphatase-2A (PP2A) [31]. Endocannabinoids also influence the cell cycle through the effects of Ras and Rho proteins on G/S transition (Supplementary Fig. S5). This pathway is highly statistically significant (*P* = 4.423e^−^^12^) and has a low false discovery rate (FDR = 5.519e^−9^) (Supplementary Fig. S5).

We confirmed these changes by real-time PCR for genes selected from each pathway (Fig. 4F), in addition to six genes from each pathway (Supplementary Figs. S2–S5). Most pathways in development and cell cycle-related genes (*Cdh1, BMP4, Cdkn1a, Ccna2, Kpnb1*, and *Rac1*) were induced by AEA, and such inductions were absent in the β-catenin KO cell line. *HIF1a* and *Bnip3*, which encode for factors in the hypoxia pathway, were also induced by AEA in wild-type but not in β-catenin KO cells (Fig. 5F). A similar pattern was evident for the hepatocyte maturation markers *Afp, Cyp1a2, Ugt2b35*, and *Oatp1b2*, which were induced by AEA in wild-type but not in β-catenin KO BMOL cells (Fig. 5G).

We next used flow cytometry to assess the effects of sustained exposure of BMOL cells to AEA (added daily for 5 days at 0.3 μM/day) on the hepatocyte differentiation markers HNF4 and albumin (Fig. 5H). AEA significantly induced the expression of both proteins in wild-type but not in Ctnnb1^CRISPR^ KO cells, indicating the β-catenin-dependence of these effects.

### Effects of AEA on mitochondrial bioenergetics in mouse HPCs

**Fig. 6:**
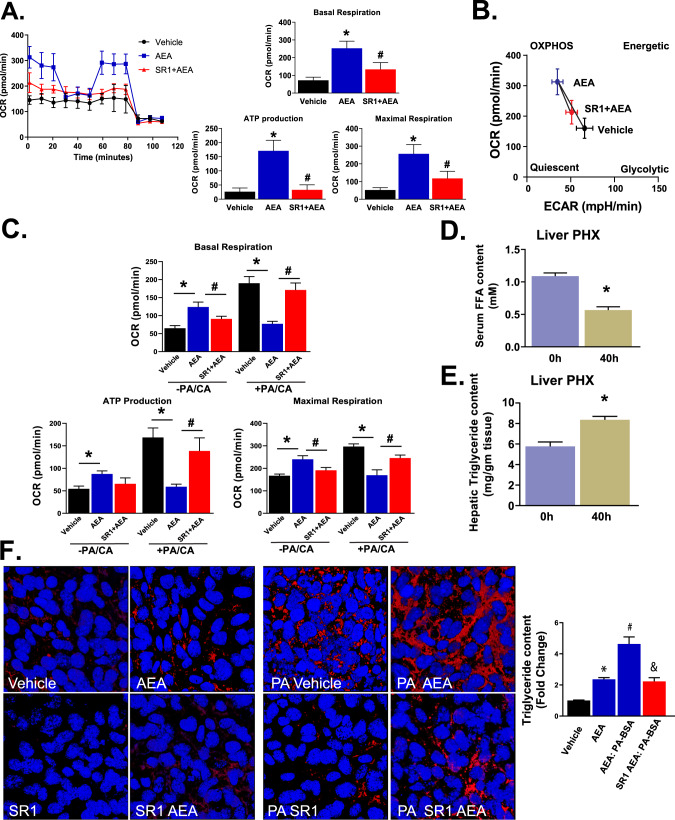
AEA regulates mitochondrial metabolism and energy homeostasis in mouse HPCs. **A** Oxygen consumption rate (OCR) under “full substrate” (see “Methods”) measured in the presence of vehicle, AEA (0.3 μM) or AEA + SR1 (1 μM). Basal and maximal respiration and ATP production were quantified. Columns and vertical bars denote means and SE, * and # indicate significant difference from the vehicle or AEA group, respectively, (*P* < 0.05, *n* = 6/group). **B** Energy map plot of OCR versus extracellular acidification rate (ECAR). **C** OCR under “limited substrate” in the absence (left 3 columns) or presence of 175 μM palmitate (right 3 columns). Parameters measured as in (**A**). Statistics and symbols as in (**A**). **D** Serum free fatty acid (FFA) content at 0 h and 40 h post-PHX); **E** Liver TG in content at 40 h post-PHX and in the remnant liver (0 h). **F** Effect of AEA on lipid droplet accumulation in the absence or presence of added palmitate and rimonabant (SR1) (left) and the quantitation on TG content (right). Columns and vertical bars denote means and SE, * indicates *P* < 0.05; *n* = 6/group.

To understand the energy requirements and metabolism of a self-renewing Axin2^+^Cnr1^+^ HPC cell line, we quantified parameters of mitochondrial respiration in BMOL cells by Seahorse instrument analysis. In an assay medium supplemented with 1 mM pyruvate, 2 mM glutamine, and 10 mM glucose (“Full Substrate”), the addition of 0.3 μM AEA significantly increased the oxygen consumption rate (OCR) under both basal and maximally stressed conditions and increased ATP production (Fig. 6A). All three effects were significantly attenuated by simultaneous exposure of the cells to SR1 (Fig. 6A). The drug treatments did not significantly modify the extracellular acidification rate (ECAR) (Fig. 6B), suggesting that the AEA-induced increase in oxidative phosphorylation (OXPHOS) was due primarily to an increase in the oxidative metabolism of glutamine and pyruvate, which, unlike glycolysis, does not result in acidification [32]. AEA elicited similar, but much smaller, effects when cells were preincubated in a limited substrate supplemented with 2.5 mM glucose and 0.5 mM carnitine (Fig. 6C, left three columns of each bar graph set). The addition of palmitate (175 μM) caused dramatic increases in both basal and maximal respiration, which were completely reversed by AEA, and the effect of AEA was again attenuated in the presence of SR1 (Fig. 6C, right three columns). Thus, AEA has opposite effects on HPC oxidative metabolism, increasing it when amino acids and glucose are used primarily as substrates and inhibits it when oxygen is used primarily for fatty acid oxidation.

PHx-induced liver regeneration is associated with transient steatosis mediated by mobilization of adipose tissue lipids [33]. Accordingly, liver triglyceride (TG) levels were significantly increased at 40 h post-PHx compared to the remnant liver (Fig. 6E), and there was a corresponding reduction in plasma-free fatty acids (Fig. 6D). AEA is known to promote TG accumulation in the liver [9, 34]. Incubation of BMOL cells with 0.3 μM AEA increased the TG content of the cells, which was greatly enhanced in the presence of exogenous palmitate and was inhibited by SR1 (Fig. 6F).

## Discussion

**Fig. 7:**
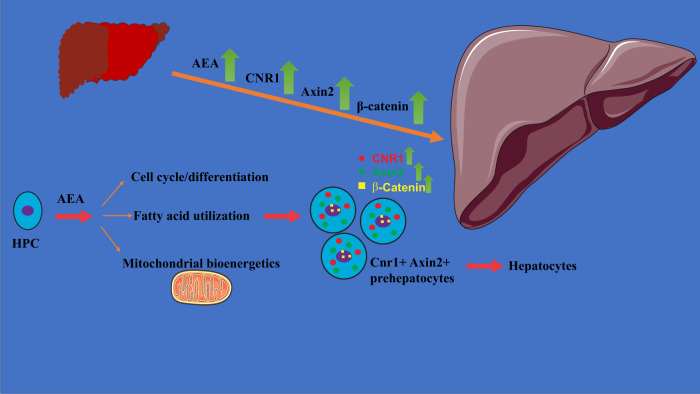
Schematic representation of similar molecular events in liver regeneration after PHx and AEA-induced cell proliferation and differentiation of hepatic progenitor cells. AEA promotes the nuclear localization of the transcription factor β-catenin, with subsequent induction of its downstream targets leading to cell cycling and differentiation. AEA also promotes mitochondrial energetics in HPCs when amino acids and glucose are readily available as substrates, but AEA inhibits it when the cells rely primarily on fatty acid oxidation. The figure was made from Servier Medical Art by Servier, licensed under a Creative Commons Attribution 3.0 Unported License.

The observations presented here reveal a hitherto unknown function of the ECS as a modulator of the proliferation and differentiation of HPCs, with relevance to the process of liver regeneration. Previously, we documented a surge in the synthesis of AEA and the expression of CB_1_R in the remnant liver following PHX in mice, with the resulting activation of CB_1_R inducing the expression of the FOXM1 transcription factor and promoting cell cycle progression in the regenerating liver [7]. Here, we have demonstrated that a distinct population of Cnr1^+^Axin2^+^ HPCs contributes to their subsequent differentiation into mature hepatocytes via signaling pathways that include β-catenin and utilizes substrate-specific cellular bioenergetics (Fig. 7).

Self-renewing Axin2^+^ HPCs are involved in the homeostatic replenishment of mature and aging hepatocytes [17]. We observed a significant increase of Axin2^+^ cells 40 h after PHX, which closely paralleled an increase in *Cnr1* expression. Furthermore, PHX induced the hepatic gene expression of β-catenin, the transcriptional regulator of Axin2, with a similar time course. The tissue levels of GSK3β protein and cyclin D1, upstream regulator and downstream target of β-catenin, respectively, were also induced following PHX. Similar to the present findings, upregulation of β-catenin and GSK3 at 5 days after PHX has been reported earlier in a rat model involving oval cells [35]. Together, these findings strongly suggest that the pro-proliferative effect of endocannabinoids following PHX is mediated via CB_1_R-dependent activation of a GSK3β-β-catenin–CyclinD–Axin2^+^ pathway in the liver.

Due to the difficulty in isolating and maintaining pure populations of primary HPCs, we used a rat and a mouse HPC cell line for further mechanistic studies to test the above hypothesis. In both cell lines, AEA-induced proliferation displayed a bell-shaped concentration-response relationship or hormesis, with the peak proliferative response observed at the low concentration of 50 nM, which is close to the affinity constant of AEA binding to the CB_1_R as observed in this study. The proliferative effect of AEA was attenuated by rimonabant, indicating its mediation via CB_1_R.

Transcriptome analyses had been previously used to uncover novel metabolic pathways in liver diseases [36, 37]. In this study, RNA-sequencing analyses revealed a comprehensive landscape of cellular pathways and networks targeted by AEA in HPCs. The most significant network processes affected by AEA were cell cycle and differentiation pathways through which AEA can influence HPC growth and simultaneously promote the maturation of HPCs into a hepatocyte lineage. AEA-regulated genes involved in cell cycle processes were confirmed by real-time PCR and include *Pcna*, *cMyc*, *Gsk3β*, *Rac1*, and *Fzr1* (hedgehog signaling), which were similarly modulated by PHX [11, 38–40]. Furthermore, some AEA-induced genes involved in cellular differentiation, such as *TgfβR1* and *Cdc42*, are also induced following PHX [41]. Notably, AEA-induced genes identified by transcriptome analyses include *Ctnnb1* (encoding β-catenin) and *Ccnd1* (encoding Cyclin D1), proteins that are known to have crucial roles in hepatocyte lineage differentiation.

Endocannabinoid-mediated Axin2^+^ cell proliferation also correlated with enhanced expression of CB_1_R and cytokeratin 19 (CK19) in both mouse and rat HPCs. CK19 is commonly used as an HPC marker, and its increased expression has also been reported in patients with hepatocellular carcinoma (HCC) [42, 43]. As for the AEA-induced increase in *Cnr1* expression, this is in agreement with earlier findings of the autoinduction of *Cnr1* expression by CB_1_R activation [44].

To understand the mechanism by which endocannabinoids induce Axin2 expression, we analyzed the expression of its transcriptional regulator β-catenin. In mouse HPCs treated with AEA, distinct mesh-like staining of β-catenin appeared to accumulate around the nuclear membrane. Staining was also observed in many cell nuclei and the plasma membrane when viewed at higher magnification. Furthermore, numerous β-catenin target genes were induced, suggesting that nuclear localization of β-catenin resulted in the activation of downstream targets. AEA-induced nuclear localization of β-catenin and induction of its target genes were also evident in rat HPCs. These data support the hypothesis that endocannabinoids activate β-catenin and its downstream targets in HPCs. β-catenin plays a pivotal role in the development of alcohol-associated liver steatosis, bile duct carcinoma and HCC [45–47]. CB_1_R activation was also reported to have a pathogenic role in these conditions [10, 11], which further supports a functional link between CB_1_R and β-catenin.

To further interrogate the link between β-catenin and endocannabinoid-induced HPC proliferation and differentiation, we generated β-catenin-deficient HPCs by CRISPR-Cas9 and analyzed the differential effects of AEA on gene expression in the KO cells and their wild-type controls using whole transcriptome analyses. Heatmap representation of those gene networks demonstrated a critical role for β-catenin in AEA-induced network processes including essential proteins, such as transcription factors, enzymes, receptors, kinases, proteases and phosphatases. Specifically, we screened for genes induced by AEA by more than 50% in wild-type but not in β-catenin KO cells. When subjected to GeneGo analyses, the most strongly induced genes fulfilling this criterion were components of cell differentiation and cell cycle-proliferation pathways. The expression pattern of these genes was further validated by real-time PCR, which supported the key role of β-catenin pathways in AEA-induced proliferation and differentiation of HPCs.

Most cell cycle and cell proliferative genes were constitutively upregulated in β-catenin KO cells, indicating loss of regulation. β-catenin interacts with many cell cycle proteins and transcription factors and its absence may trigger compensatory mechanisms unrelated to its regulatory pathways. Thyroid hormone receptor β agonizts also induce hepatocyte proliferation in mice during liver regeneration and the same β-catenin pathway is involved [48].

Our transcriptome data indicated significant activation of both β-catenin signaling and TGF-β receptor signaling pathways by AEA. This suggests that during liver regeneration, endocannabinoids not only promote cell cycle progression but also stimulate the maturation of HPCs. Previously, β-catenin was implicated in lineage reprogramming of hepatic cells in the endoderm of *Xenopus* embryos [49]. CB_1_R signaling plays a critical role in liver development in *Danio rerio* (zebrafish) embryos [50]. Transcriptome analyses suggest that metabolic remodeling occurs during the process of liver regeneration [51]. Another unexpected and interesting observation in the present study is the AEA-induced, β-catenin-mediated regulation of inflammation and cell cycle regulation of interleukin expression in HPCs. Immune cells play a critical role in liver regeneration [52], during which premature hepatocytes release a variety of interleukins, thus behaving similar to immune cells in this process.

To further dissect the molecular machinery involved in the initiation of differentiation, we incubated wild-type and β-catenin KO mouse HPCs with AEA for five days and estimated the cellular levels of the hepatocyte markers HNF4 and albumin [53, 54], by immunohistochemical staining followed by flow cytometry. The observed AEA-induced increase of HNF4 and albumin levels in wild-type cells but not in β-catenin KO HPCs indicates that CB_1_R activation promotes maturation of cells in the hepatocyte lineage. AEA likely has a similar role in the regenerating liver, as suggested by its marked and sustained increase in the remnant liver following PHx [7, 8]. Because of the link between CB_1_R activation and β-catenin expression, we may infer that β-catenin is responsible for the endocannabinoid-mediated initiation of hepatocyte maturation. This, of course, does not negate the possible role of numerous other paracrine factors in the hepatocyte maturation process.

There is a physiological gradient in the liver where the oxygen tension of 60–65 mmHg in the periportal region declines to 30–35 mmHg in the perivenous zone 3 regions [55]. Axin2^+^ HPCs are preferentially localized in the perivenous region where the hypoxic milieu promotes their maintenance in the quiescent state, by analogy to hematopoietic stem cells localized in hypoxic niches of the bone marrow [56]. Under these conditions, HPCs must rely on anaerobic glycolysis for ATP production for their self-renewal, similar to hematopoietic stem cells [57]. Hypoxia induces the expression of HIF1α and we found that AEA induces *HIF1α* and *Bnip3* expression in a β-catenin-dependent manner. Wnt-β-catenin signaling in the liver was shown to promote cell proliferation by increasing glutamine metabolism [58]. HIF1α in complex with HIF1β is also known to promote the expression of multiple glycolytic genes [59]. Together, these observations suggest that in the early stages following PHx, characterized by hypoglycemia and reduced availability of free fatty acids due to an increase in de novo lipogenesis [60], the energy requirements of AEA-induced HPC proliferation is provided by increased glutamine metabolism and, to a lesser degree, by glycolysis.

The most striking effect of AEA was modulation of mitochondrial energy metabolism. Mitochondrial energy metabolism plays a critical role in immune cell differentiation and cell cycle [61, 62]. In sharp contrast to the AEA-induced increase in oxygen consumption rate (OCR) derived from glutamine, pyruvate, and glucose substrates, AEA robustly inhibits the palmitate-induced increase in OCR. This effect may not be unexpected in view of the well-established role of CB_1_R activation to inhibit fatty acid oxidation in a variety of tissues, including liver [9, 63], brown fat [64], kidney [65], sperm cells [66], and ghrelin-producing cells of the stomach mucosa [67]. As discussed above, in the late phases of liver regeneration activation of CB_1_R by AEA promotes the terminal differentiation of hepatocytes, which would require a brake on the energetic support of cell proliferation. Inhibition of OXPHOS may also be a mechanism to preserve the self-renewing potential of HPCs and prevent their senescence and depletion that can result from uncontrolled proliferation.

A limitation of this study is that primary HPCs were not studied due to technical challenges of indistinguishable heterogeneous population of HPCs and hepatocytes in the PHx model. However, the insight that Axin2^+^Cnr1^+^ HPCs have a unique endogenous cannabinoid-modulated role in hepatic cell proliferation, maturation and metabolic remodeling during liver regeneration. These findings could potentially be leveraged to improve liver regeneration and thus treat acute liver failure or organ dysfunction in chronic liver disease.

## Methods

### Cell culture

Mouse BMOL hepatocyte progenitor cells and permission for their genetic manipulation were obtained from Dr. George Yeoh (The University of Western Australia). Cells were cultured in Williams’ E medium containing 5% FCS, antibiotics, glutamine, 20 ng/mL epidermal growth factor (BD Biosciences) and 30 ng/mL human insulin (Sigma-Aldrich) [68].

The rat LE2 non-tumorigenic hepatocyte progenitor cell line was generated by Dr. N. Fausto’s lab [69] and was kindly provided by Drs. Jean Campbell and Renay Bauer (University of Washington). Cells were maintained in a 1:1 mix of DMEM (Thermo Fisher Scientific) and Ham’s F10 supplement (Thermo Fisher Scientific) with 10% fetal bovine serum (Thermo Fisher Scientific), insulin at 1 µg/mL (Sigma), hydrocortisone (0.5 µg/mL, Sigma), and gentamicin (10 µg/mL, Thermo Fisher Scientific). Cell proliferation method provided in the supplemental section.

All other methods are provided with details as supplemental materials.

## Supplementary information

Supplemental Method

Supplemental Figure legends

Figure S1

Figure S2

Figure S3

Figure S4

Figure S5

RNASEQ data

Original Data File

## Data Availability

RNA-Seq data are provided in RPKM values as a supplemental file.
