# Supplementary material for: The endocannabinoid system promotes hepatocyte progenitor cell proliferation and maturation by modulating cellular energetics
Source: Cell Death Discov. 2023 Mar 25;9:104. doi: 10.1038/s41420-023-01400-6 (PMC10039889; doi:10.1038/s41420-023-01400-6)
Supplement: Supplementary file 1 — Supplemental Method [file 41420_2023_1400_MOESM1_ESM.docx]

**The endocannabinoid system promotes hepatocyte progenitor cell proliferation and maturation by modulating cellular energetics.**

Bani Mukhopadhyay *et al.*

**Supplemental Method**

**Animals**

All procedures were approved by the NIAAA Institutional Animal Care and Use Committee and were performed by the National Institutes of Health Guide for the Care and Use of Laboratory Animals. C57BL/6J mice were from Jackson Laboratories. Male mice 10–12 wk of age were used in all experiments as described earlier ^1^. CB_1_R^−/−^ and CB_1_R^+/+^ littermates were obtained by breeding heterozygotes that had been backcrossed to a C57BL/6J background, as described ^2^. All animals were randomised to the treatment groups without considering any other variable. Blinding were done during animal experiment and histologic studies.

**Liver Regeneration Mouse Model**

Two-thirds partial hepatectomy was performed as described ^3^ and used as a model of liver regeneration. The removed liver tissue was snap-frozen in liquid nitrogen and used for analyses as 0 h control samples. The animals were killed by decapitation under deep anesthesia at 40 h after surgery, and the remnant liver was collected and snap-frozen in liquid nitrogen or placed in 10% formalin for histology. Some tissue samples were placed in O.C.T compound, kept frozen and processed for RNAScope analyses.

**Liver histology**

Paraffin-embedded liver tissues were sliced with microtome with thin section (5µm). The sections were deparaffinized followed by antigen retrieval using citrate buffer as described earlier ^4^.

**Genome Engineering of BMOL Cells**

Knocking out the β-catenin gene was achieved by precision genome editing using the CRISPR-Cas9 system ^5^. Guide gRNA (AGCTACTTGCTCTTGCGTGA) was incorporated into a lentivirus with plasmid plentGuide-catenin beta 1 gRNA1-puro (#U3309CA180, GenScript) and transfected into BMOL cells (efficiency >60%). After screening for knock-out cells, single clones with the desired β-catenin gene knock-out were selected and further characterized by sequencing (GenScript). The final BMOL clone with a 2-bp deletion (Ctnnb1^CRISPR^) in the coding region of the gene encoding β-catenin was cultured in the continued presence of blasticidin and puromycin to maintain selection pressure.

**RNA Isolation**

Total RNA was isolated from whole liver or from HPC cell lines using Direct-zol RNA Kits according to the manufacturer's instructions (Zymo Research).

**Deep RNA-Sequencing**

For RNA sequencing experiments, RNA samples were pooled from three independent experiments using wild-type or Ctnnb1^CRISPR^ BMOL cell lines treated with vehicle or anandamide at 300 nM. Samples were processed as described in supplemental method and sequenced using the Ion Torrent Proton instrument. All four pooled libraries contained up to 33 million reads per sample, mapped to the mouse mRNA transcriptome.

**Analyses of RNA-Sequencing Data**

After Ion Torrent sequencing was completed, BAM files containing sequence reads were imported into the CLC Genomics Workbench (version 11.0, Qiagen) for mapping and downstream analysis. Data were provided in RPKM values as supplemental file.

**Pathway Analyses of RNA-Sequencing Data**

Files with gene id and corresponding RKPM values were uploaded to GeneGo Metacore (Thomson Reuters) and significantly affected network processes and pathways were determined.

**Measurement of Real-Time Oxygen Consumption and Extracellular Acidification Rates**

Real-time oxygen consumption rate (OCR) and extracellular acidification rate (ECAR) were measured using Seahorse XF Cell Mito Stress Test Kit (#103708-100) in a Seahorse XF analyzer (Seahorse Bioscience) according to the manufacturer’s instructions. Briefly, the cartridge plate was hydrated with XF calibrant buffer and incubated overnight (37°C, CO_2_-free). The assay mediums with the full substrate (XF base DMEM medium #103575-100, Agilent, containing 1 mM pyruvate, 2 mM glutamine, and 10 mM glucose) or limited substrate (FAO Assay Medium composed of KHB buffer [111 mM NaCl, 4.7 mM KCl, 1.25 mM CaCl_2_, 2 mM MgSO_4_, 1.2 mM NaH_2_PO4] supplemented with 2.5 mM glucose, 0.5 mM carnitine, and 5 mM HEPES adjusted to pH 7.4 at 37°C) were prepared immediately before use. To limited substrate media, fatty acids were added using the XF Palmitate-BSA FAO Substrate (#102720-100, Agilent), for a final concentration of 175 µM palmitate. Cells from four different flasks were plated with an equal number of cells (10^5^) in four wells of the Seahorse plate. Cells were deprived of supplemental growth factors for 5 hours. Cells were maintained for 1 hour in either full substrate or limited substrate assay media before assays. All media contained vehicle or anandamide with or without SR1, as described in the legend of figure 7.

**Cell proliferation**

Cell proliferation was quantified in living cells using live cell water-soluble tetrazolium salt (WST-8) dye and Cell Counting Kit-8 (CCK-8, Dojindo Molecular Technologies, Inc.). Cell proliferation was also assayed by incorporation of BrdU (5-bromo-2'-deoxyuridine), as detected by using BrdU In-Situ Detection Kit (550803, BD Bioscience). Image data were quantified using Image J (NIH) software by analyzing particles with a circularity of greater than 0.2.

**Pathway Analyses of RNA-Sequencing Data**

Files with gene id and corresponding RKPM values were uploaded to GeneGo Metacore (Thomson Reuters) and significantly affected network processes and pathways were determined. Individual pathways were exported along with detailed statistical parameters. Each pathway with significant changes in gene expression (either more than 20% greater or lower in anandamide-teated compared to vehicle-treated control samples) was further analyzed by ,software R (<https://www.r-project.org/>), using the heatmap.2 function in the package “gplots” (<https://www.rdocumentation.org/packages/gplots/versions/3.0.1>). Networks and subcellular interactome (Fig 7B-D; Figure S1A) were selected based on the enrichment profile of the pathways sorting with statistically significant *P*-value, low false discovery rate (FDR), Z score and g-score. Complete transcriptome data without any filter were uploaded for these analyses.

Representative pathway maps (Figure S4-7) were selected based on enrichment profile of the pathways with sorting statistically significant p-value, low false discovery rate (FDR) and relevant to cell cycle or differentiation network processes. In addition, input transcriptome data were filtered based on two essential criteria: 1) induction by 50% or more by anandamide and 2) loss of such induction in β-catenin KO cells. A graphic legend of all symbols and pathway parameters details with color guide can be found at GeneGo portal (https://portal.genego.com/legends/MetaCoreQuickReferenceGuide.pdf)

**Statistical Analyses of Gene Expression Data**

Statistical analyses were performed in MetaCore Clarivate analytic, as detailed in their website and briefly summarized below.

In MetaCore, algorithms such as “Analyze networks” were used to build a set of network modules associated with input data of gene expressios). *P*-value calculations are based on hypergeometric distribution. MetaCore also uses *P*-value calculation to evaluate network’s relevance to Gene Ontology biological processes classification.

False discovery rate (FDR) is derived from statistical procedures where multiple statistical tests are conducted simultaneously with the intention to limit the number of. possible number of type I errors. For example, an FDR threshold at the level of 0.05 means that the list of significant processes or objects will contain no more than 5% of false positive results.

The Z-score ranks the subnetworks of the analyze network algorithm with regard to their saturation with genes from the experiment. A high Z-score means the network is highly saturated with genes from that particular experiment.

The G-score modifies the Z-score based on the number of canonical pathways used to build the network. If a network has a high G-score, it is saturated with expressed genes (from Z-score), and it is enriched in canonical pathways.

**Real-time PCR**

Real-time PCR was performed using individual primer assay kits (Qiagen) as provided in the below table and syber green (Vita Scientific). The experiments were performed using an ABI 7500 system and analyzed by SDS software. Each amplified sample was analyzed for homogeneity using dissociation curve analysis. Relative quantification was performed using the comparative CT method.

**RNA *In Situ* Hybridization**

Frozen liver samples sectioned by cryotome (10µm) or cell lines grown on glass bottom dishes were processed for RNA *in situ* detection using the RNAscope® Multiplex Fluorescent assay according to the manufacturer’s instructions (Advanced Cell Diagnostics). RNAscope probes used were: Axin2 (RNAscope® Probe- Mm-Axin2, 400331), CNR1 (RNAscope® Probe- Mm-Cnr1-O1-C2, #457341-C2), CK19 (RNAscope® Probe- Mm-CK19-C3, #300031-C3). Cell nuclei were stained with DAPI provided with the kit. The fluorescent images were taken by confocal microscope (LSM 700, Carl Zeiss) using oil immersion objective 40X or 100X. The scale bar was provided with all images.

**Immunofluorescence and confocal microscopy**

Cells were grown on cover glass in 12-well dishes or 12-well glass bottom dishes. Cells were synchronized in serum-free media for at least 6 h followed by treatments as described. Cells were fixed with 4% paraformaldehyde followed by permeabilization with BD Cytofix/Cytoperm™ Kit (#554714, BD Bioscience). The cells were stained with antibodies against β-catenin (#9582, Cell Signaling Technology) followed by secondary antibodies conjugated to Alexa Fluor 594 (#A-11012, Life technologies) and Alexa Fluor 488(# A-11034 , Life technologies) respectively. Cell nuclei were stained with Hoechst 33342 (H3570, Thermo Fisher Scientific). After mounting with VECTASHIELD Antifade Mounting Medium (H-1000, Vector Laboratories), images were taken in LSM 700 confocal microscope (Carl Zeiss) with an oil immersion objective 40X or 100X. The scale bar was provided with all images.

**CHIP assay**

CHIP assays were performed as described earlier^6^. CHIP quality Axin 2 primer and β-catenin antibody were commercially obtained (#8973 and #8480; Cell Signaling Technologies).

**Flow cytometry**

After treatment, cells were processed by fixing and permeabilization with BD Cytofix/Cytoperm™ Kit (#554714, BD Biosciences) and intracellular staining was performed using primary antibodies to albumin (#ab106582, Host species: Chicken, Abcam) and HNF4α (#3113, Host species: Rabbit, Cell Signaling Technology). Secondary conjugate to Alexa Fluor 488 (# A-11034, Life technologies) and Alexa Fluor 647 (#A32933, Life Technologies) was used for albumin and HNF4 staining respectively. Cells were analyzed with flow cytometer BD FACSCALIBUR (BD Biosciences) and the data were analyzed by flowjo software 10.5 (Flowjo LLC).

**Western blotting**

Liver samples and pelleted cells were homogenized using Precellys homogenizer with ceramic beads and cells were lysed using T-PER buffer (Pierce) containing protease inhibitor (Roche) and phosphatase inhibitor mixture set I (Calbiochem). Equal amounts (25 - 50 μg/lane) were fractionated on Criterion 4–12% BisTris gel (Bio-Rad) and transferred onto a nitrocellulose membrane using a TURBO apparatus (Bio-Rad). Blocking was carried out with Blocking buffer (Pierce). The primary antibodies against GSK3β (#9327, Cell Signaling Technology), CyclinD1 (#2978, Cell Signaling Technology), Tubulin (#ab7291, Abcam), were added according to the manufacturers’ recommended dilution in a buffer (2-5% BSA and 1 X PBS containing 0.1% Tween 20) for overnight incubation at 4°C. After three washes in PBS-0.1% Tween 20, secondary HRP conjugate (PerkinElmer) was added, followed by three washes in the same buffer. Signals in blots were detected with Supersignal West Pico chemiluminescent substrate (Pierce) and developed using Eastman Kodak Co. Biomax film (PerkinElmer). Autoradiograms were scanned and quantified using Image J after binary conversion (NIH). All blots were normalized to Tubulin level.

**Statistical Analyses**

Results are reported as mean ± SE. Statistical significance among groups was determined by one-way ANOVA followed by post hoc Newman–Keuls test using GraphPad Prism 4.3 software. Probability values of P < 0.05 were considered significant. Statistical significance between two groups was determined by the two-tailed unpaired non-parametric Student *t*-test.

1. Mukhopadhyay B, Cinar R, Yin S, Liu J, Tam J, Godlewski G*, et al.* Hyperactivation of anandamide synthesis and regulation of cell-cycle progression via cannabinoid type 1 (CB1) receptors in the regenerating liver. *Proc Natl Acad Sci U S A* 2011, **108**(15)**:** 6323-6328.

2. Zimmer A, Zimmer AM, Hohmann AG, Herkenham M, Bonner TI. Increased mortality, hypoactivity, and hypoalgesia in cannabinoid CB1 receptor knockout mice. *Proc Natl Acad Sci U S A* 1999, **96**(10)**:** 5780-5785.

3. Mitchell C, Willenbring H. A reproducible and well-tolerated method for 2/3 partial hepatectomy in mice. *Nat Protoc* 2008, **3**(7)**:** 1167-1170.

4. Mukhopadhyay B, Schuebel K, Mukhopadhyay P, Cinar R, Godlewski G, Xiong K*, et al.* Cannabinoid receptor 1 promotes hepatocellular carcinoma initiation and progression through multiple mechanisms. *Hepatology* 2015, **61**(5)**:** 1615-1626.

5. Ran FA, Hsu PD, Wright J, Agarwala V, Scott DA, Zhang F. Genome engineering using the CRISPR-Cas9 system. *Nat Protoc* 2013, **8**(11)**:** 2281-2308.

6. Mukhopadhyay B, Liu J, Osei-Hyiaman D, Godlewski G, Mukhopadhyay P, Wang L*, et al.* Transcriptional regulation of cannabinoid receptor-1 expression in the liver by retinoic acid acting via retinoic acid receptor-gamma. *J Biol Chem* 2010, **285**(25)**:** 19002-19011.
