# Supplementary material for: The endocannabinoid system promotes hepatocyte progenitor cell proliferation and maturation by modulating cellular energetics
Source: Cell Death Discov. 2023 Mar 25;9:104. doi: 10.1038/s41420-023-01400-6 (PMC10039889; doi:10.1038/s41420-023-01400-6)
Supplement: Supplementary file 2 — Supplemental Figure legends [file 41420_2023_1400_MOESM2_ESM.docx]

**Legends for Supplemental Figures**

**Figure S1. Effect of AEA on the proliferation and co-expression of Axin2, CNR1 and CK19 (Krt19) mRNA in rat HPCs (LE2 cells). A.** The effect of 50-300 nM anandamide (AEA) versus vehicle on the proliferation of LE2 cells. **B.** BrdU incorporation in LE2 cells as detected by fluorescence confocal microscopy in response to the indicated concentrations of AEA with or without the CB1R antagonist SR1 (rimonabant). **C.** RNAscope analyses of the effect of AEA (300 nM) on the expression of Axin2, Cnr1 and CK19 in LE2 cells. Yellow arrows in panel A illustrate co-expression of the 3 mRNAs in the same cell. Co-expression is also illustrated by an overlay.

**Figure S2.** **Effect of anandamide in WNT β-catenin signaling in the nucleus as development process in the network of hepatic progenitor cells**. RKPM values from transcriptome data for β-catenin and Axin2 are provided along with graphical presentation for clarity.

**Figure S3. Effect of anandamide on TGF-β receptor signaling.** TGF-β 1 initiates signaling by binding to and bringing together type I (β-catenin dependent upregulation verified by RT-PCR) and type II receptor serine/threonine kinases (TGF-β receptor type I and II) on the cell surface. TGF-β1 also activates p38 MAPK and mitogen-activated protein kinase kinase 1 and 2 (MEK1 and MEK2) in differentiation. Four genes (PAI1, IKK-alpha, MEK1 and TGF-b R1) from this specific pathway were verified by real-time PCR (n=3/groups, * statistically significant p<0.05 compared to vehicle, # statistically significant p<0.05 compared to AEA treated WT cell line).

**Figure S4. Effect of anandamide on G1 to S transition in cell cycle.** Both TGF-β1 and TGF-β2 participate in the regulation of the G1/S checkpoint. TGF-β factors induce an association of its receptor with the regulatory subunit of protein phosphatase-2A (PP2A). Four genes (PP2A, CDK2, SP1, p27KIP1) from the pathway were further verified by real-time PCR (n=3/groups, *P<0.05 compared to vehicle).

**Figure S5. Effect of endocannabinoid in cell cycle through the influence of Ras and Rho proteins on G1S Transition.** H-Ras (Harvey rat sarcoma viral oncogene homolog) activates G1/S transition and cell proliferation mainly by v-raf-1 murine leukemia viral oncogene homolog 1 (c-Raf-1)/Mitogen-activated protein kinase kinases 1/2 (MEK1/2)/ Mitogen-activated protein kinases 3/1 (ERK1/2) cascade. Genes (CDC42, ROCK1, RB1 and GSK3-beta) were verified by real-time PCR (n=3/groups, * statistically significant p<0.05 compared to vehicle; # statistically significant p<0.05 compared to AEA treated WT cell line).
