# Supplementary figures and images for: The endocannabinoid system promotes hepatocyte progenitor cell proliferation and maturation by modulating cellular energetics
Source: Cell Death Discov. 2023 Mar 25;9:104. doi: 10.1038/s41420-023-01400-6 (PMC10039889; doi:10.1038/s41420-023-01400-6)

**A.**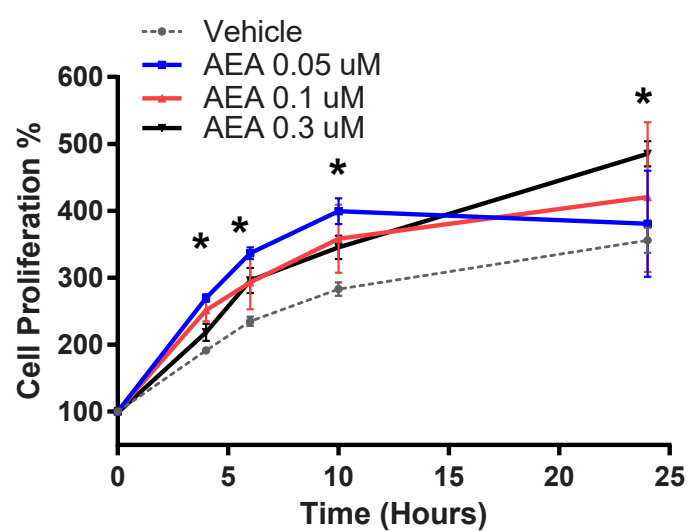**B.**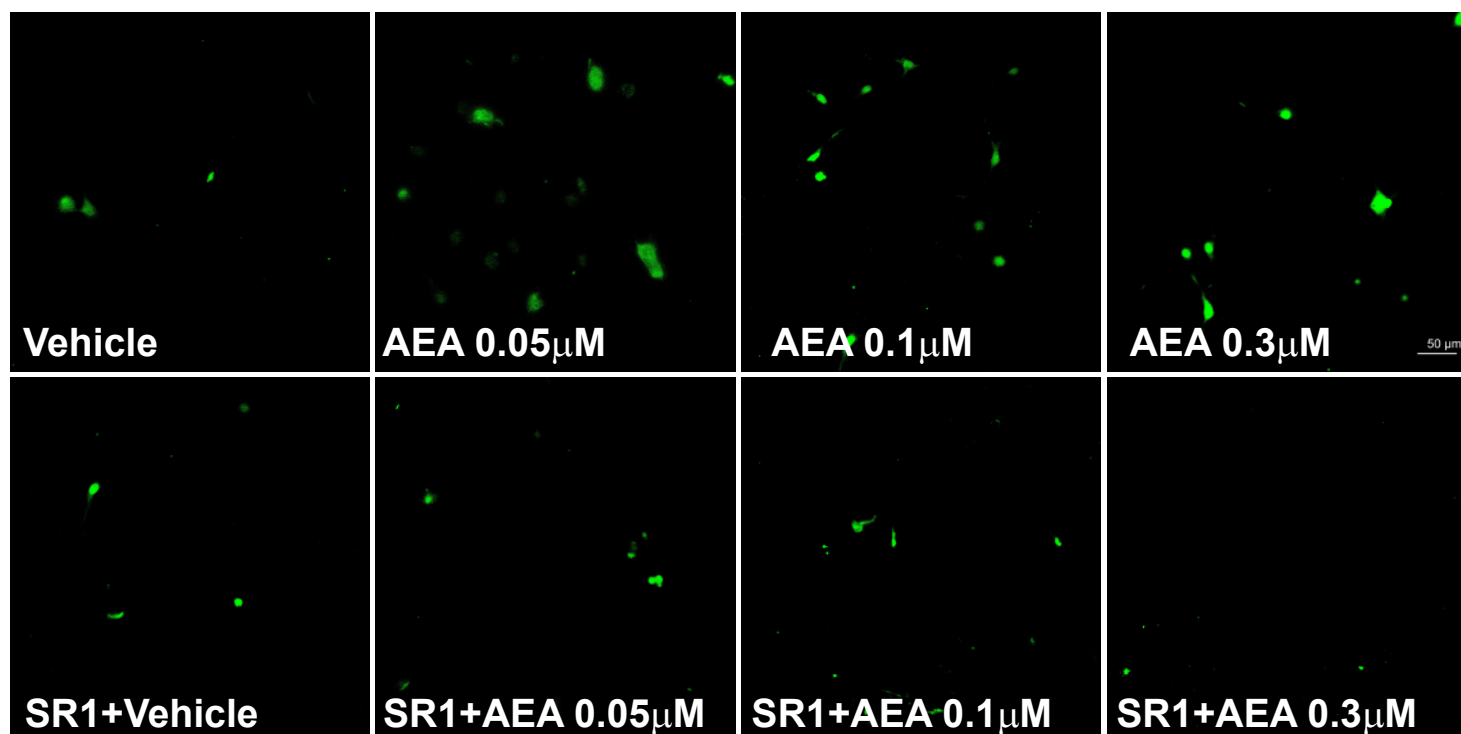**C.**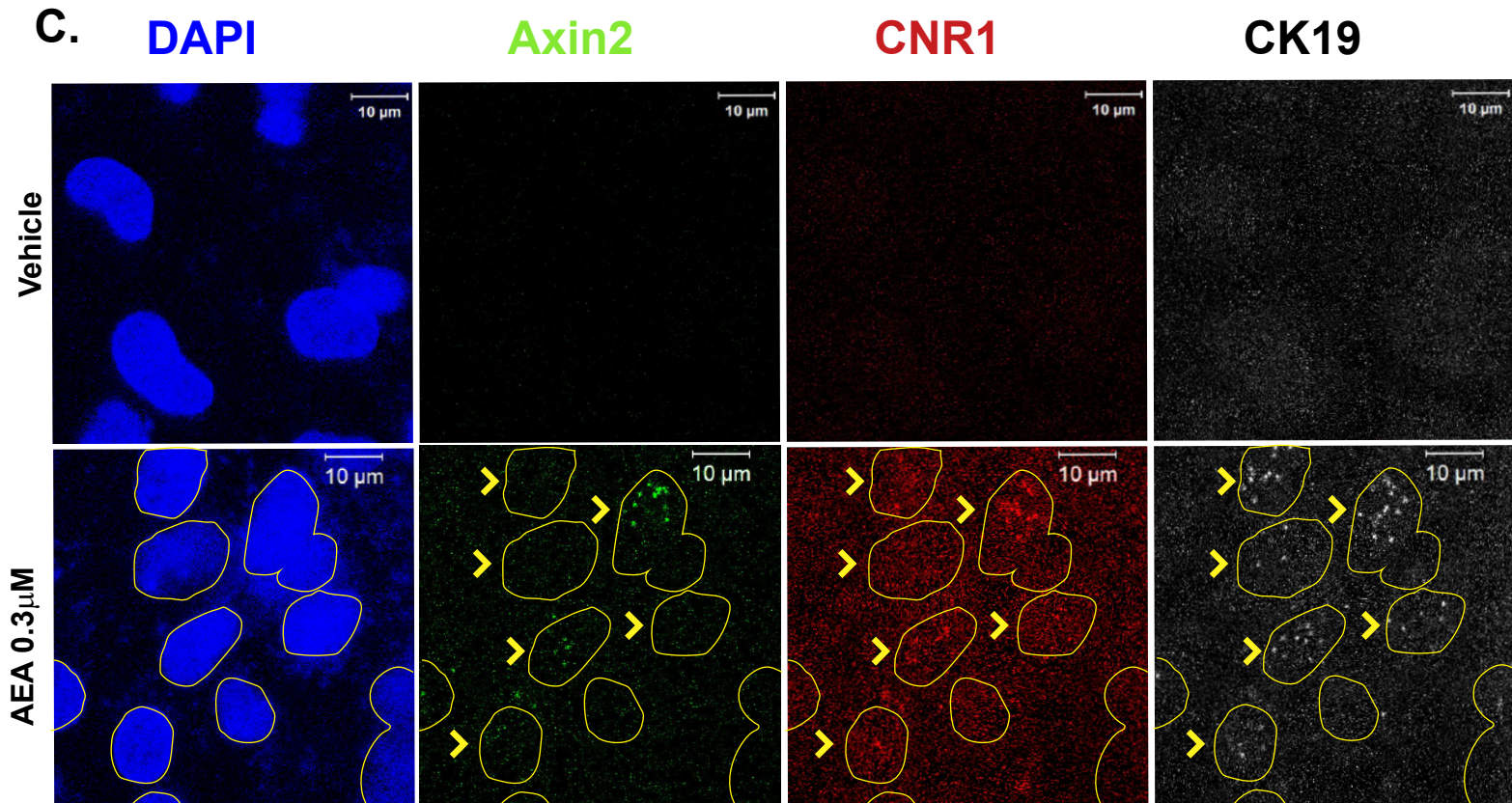**LE2****Figure S1**

Supplement: Supplementary file 3 — Figure S1 [file 41420_2023_1400_MOESM3_ESM.pdf]

p value =  $1.337e^{-4}$   
FDR =  $7.905e^{-4}$

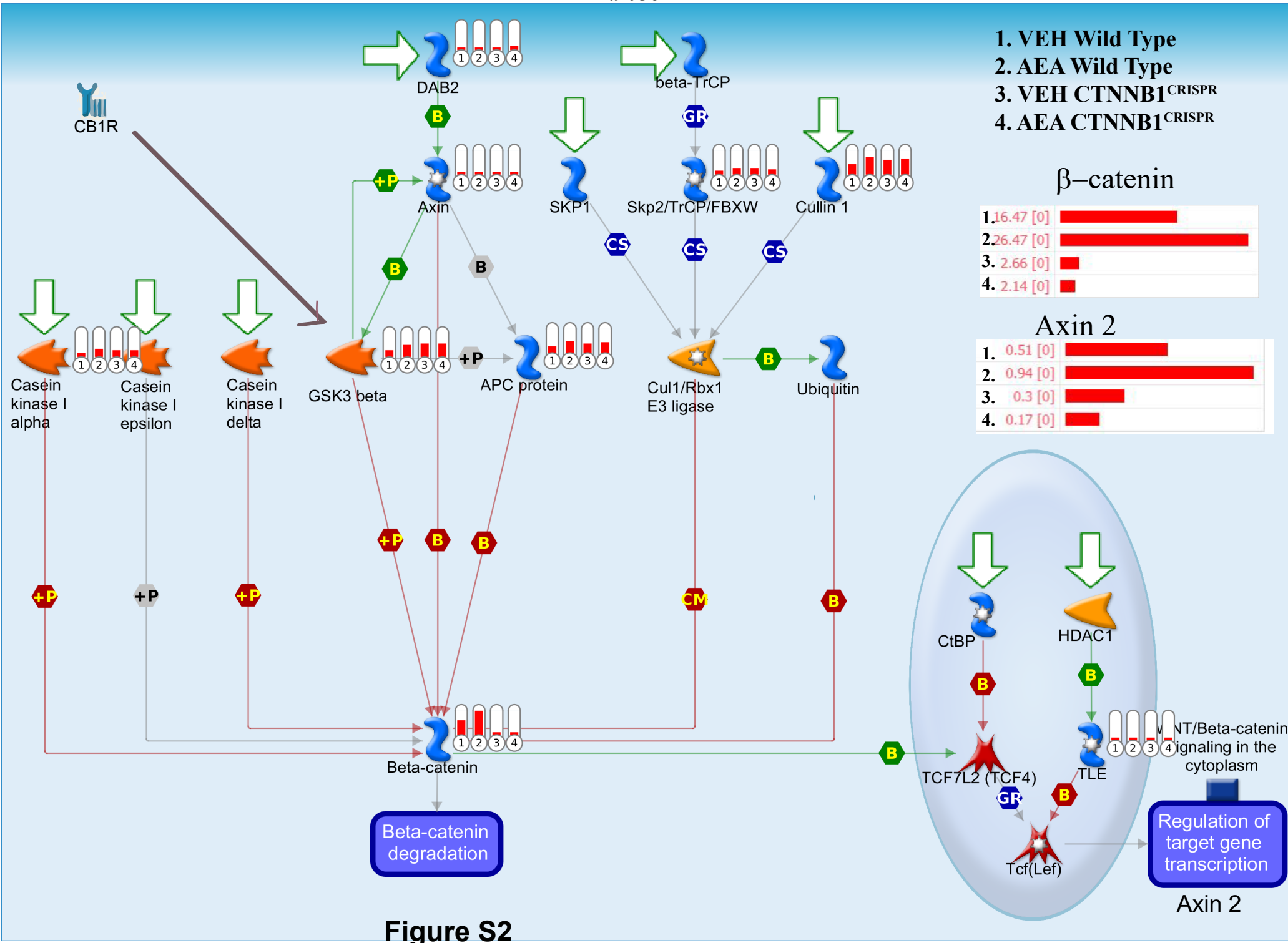

Supplement: Supplementary file 4 — Figure S2 [file 41420_2023_1400_MOESM4_ESM.pdf]

Figure 1C : original western blots

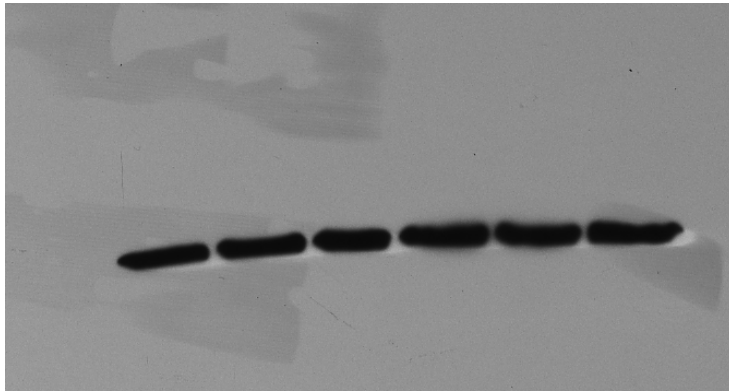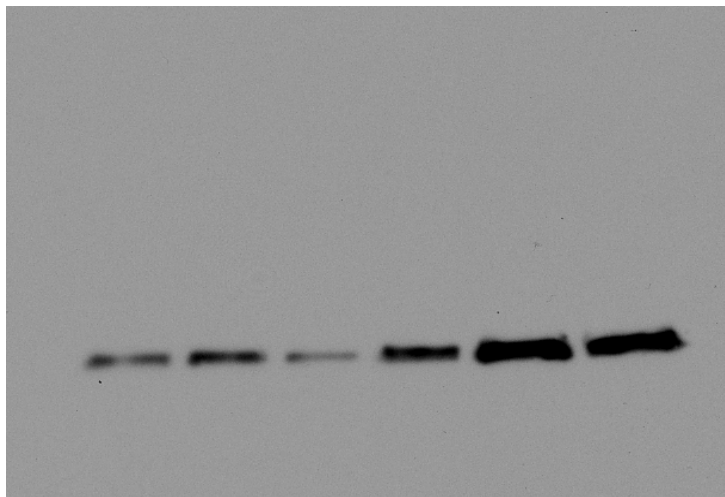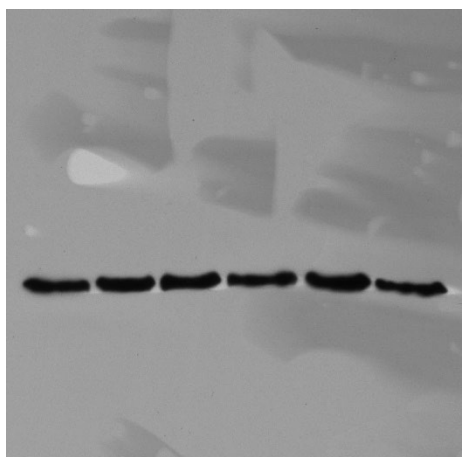

Figure 3D

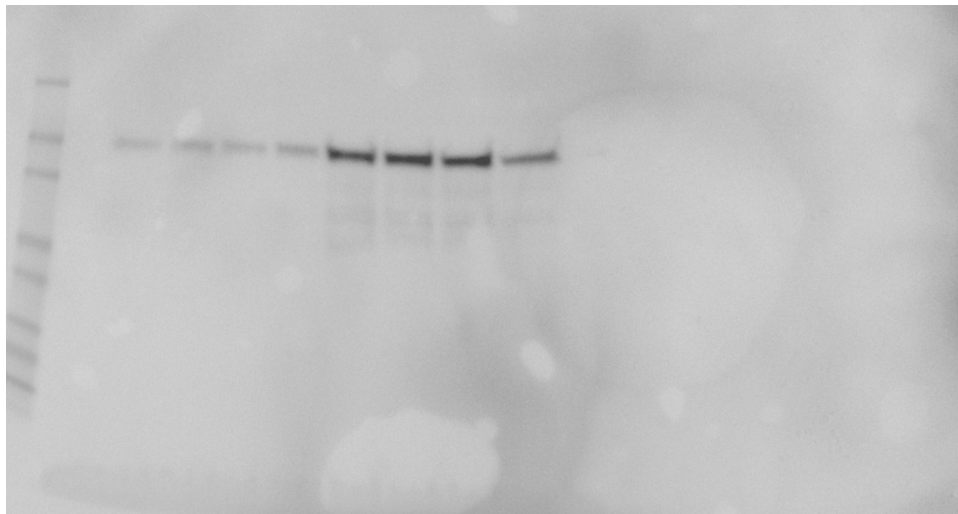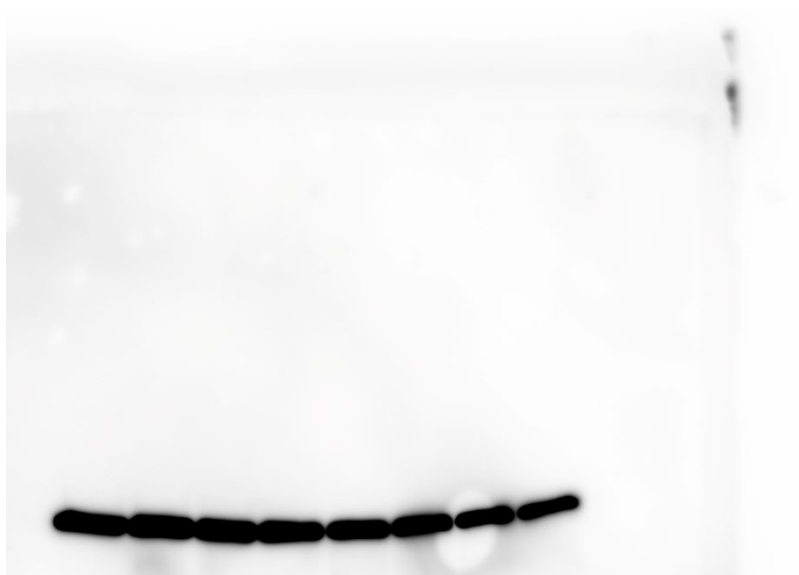

Supplement: Supplementary file 9 — Original Data File [file 41420_2023_1400_MOESM9_ESM.pdf]
