# Supplementary material for: The endocannabinoid system promotes hepatocyte progenitor cell proliferation and maturation by modulating cellular energetics
Source: Cell Death Discov. 2023 Mar 25;9:104. doi: 10.1038/s41420-023-01400-6 (PMC10039889; doi:10.1038/s41420-023-01400-6)
Supplement: Supplementary file 5 — Figure S3 [file 41420_2023_1400_MOESM5_ESM.pdf]

p value = 6.664e-9
FDR = 6.010e-7

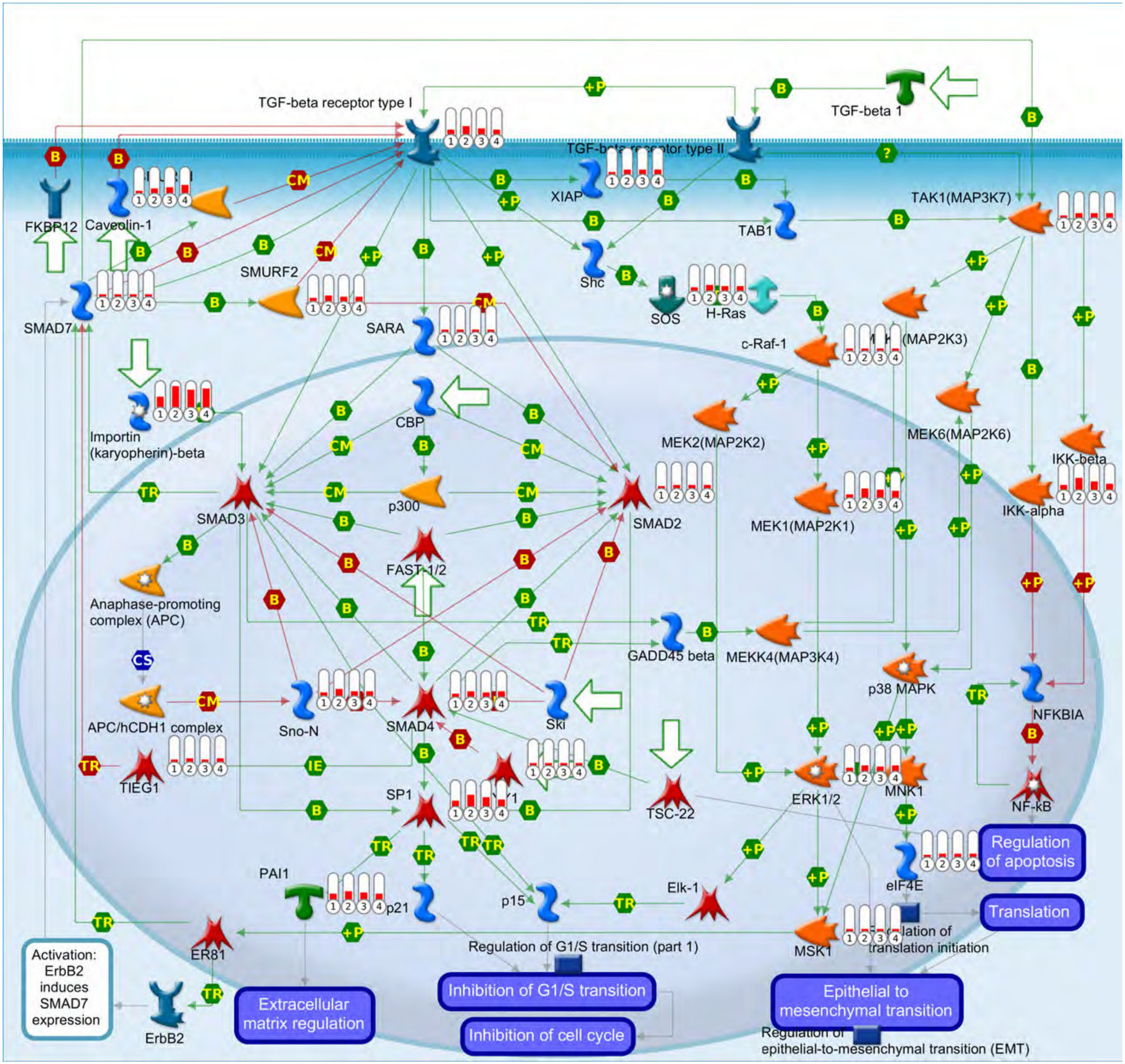

1. VEH Wild Type
2. AEA Wild Type
3. VEH CTNNB1^{CRISPR}
4. AEA CTNNB1^{CRISPR}

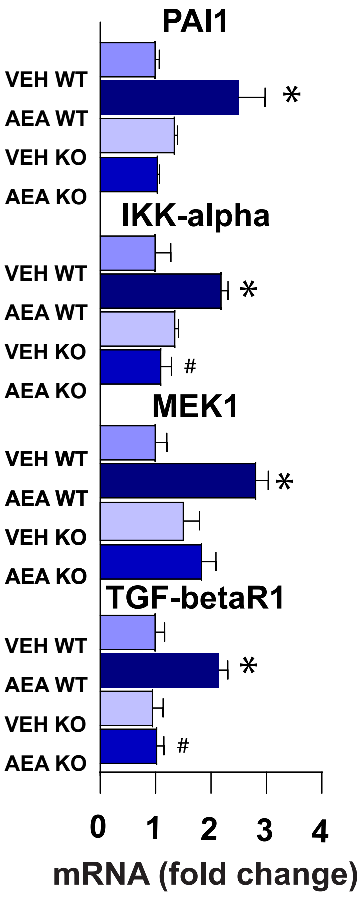

Figure S3
