# Supplementary material for: The endocannabinoid system promotes hepatocyte progenitor cell proliferation and maturation by modulating cellular energetics
Source: Cell Death Discov. 2023 Mar 25;9:104. doi: 10.1038/s41420-023-01400-6 (PMC10039889; doi:10.1038/s41420-023-01400-6)
Supplement: Supplementary file 6 — Figure S4 [file 41420_2023_1400_MOESM6_ESM.pdf]

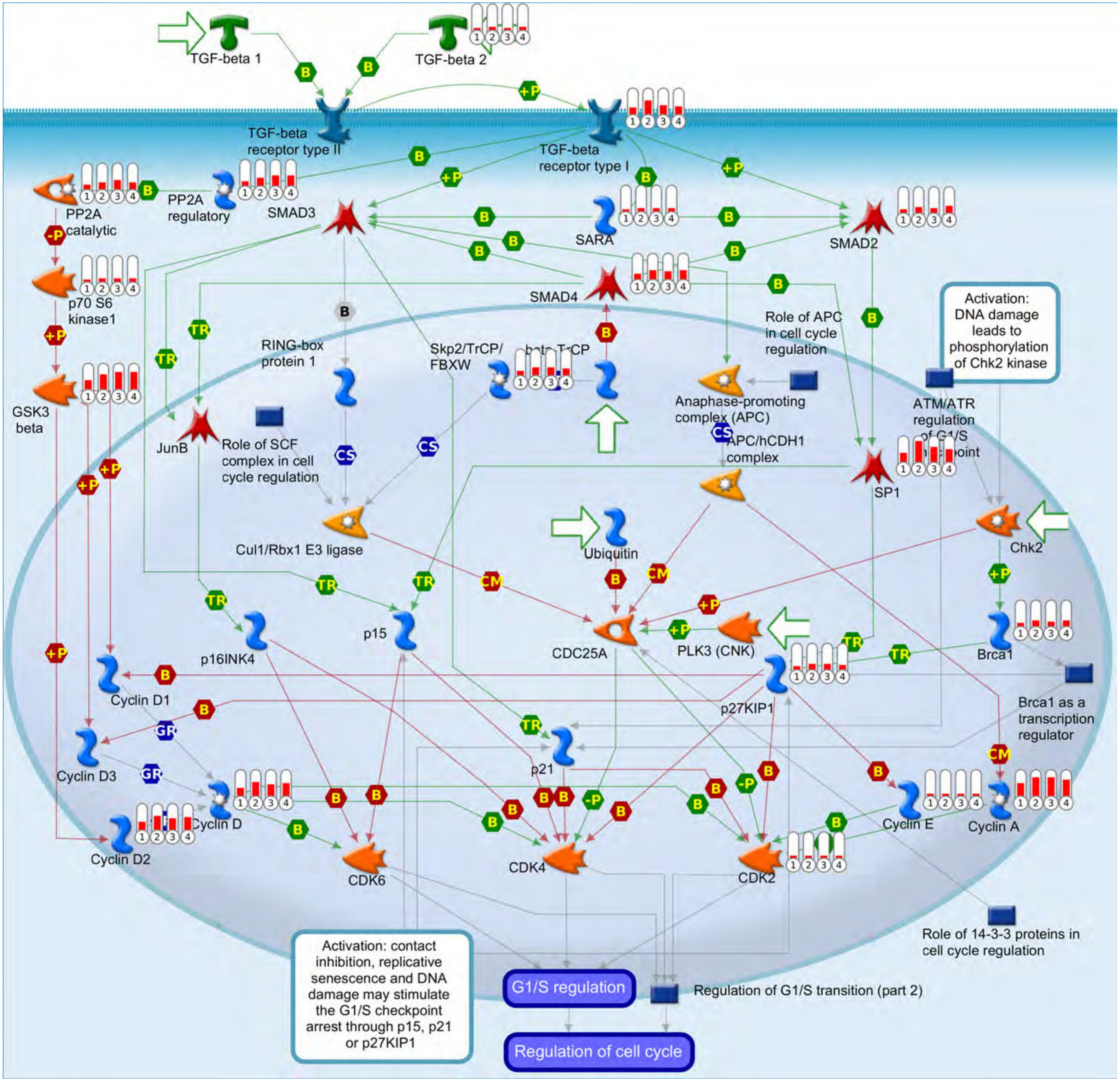

p value = 1.823e^{-8}
FDR = 1.286e^{-6}

1. VEH Wild Type
2. AEA Wild Type
3. VEH CTNNB1^{CRISPR}
4. AEA CTNNB1^{CRISPR}

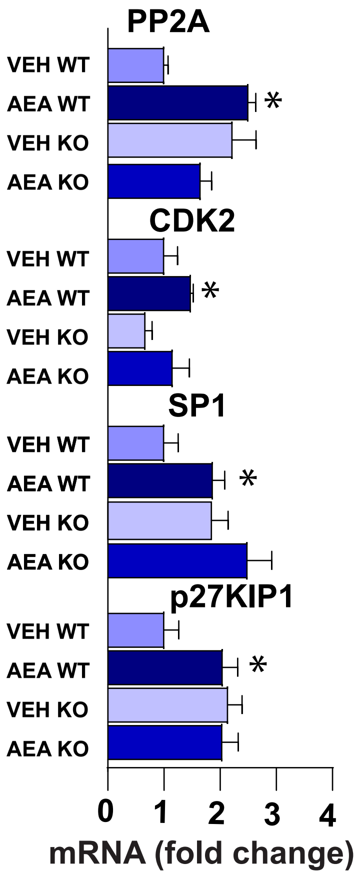

Figure S4
