# Supplementary material for: The endocannabinoid system promotes hepatocyte progenitor cell proliferation and maturation by modulating cellular energetics
Source: Cell Death Discov. 2023 Mar 25;9:104. doi: 10.1038/s41420-023-01400-6 (PMC10039889; doi:10.1038/s41420-023-01400-6)
Supplement: Supplementary file 7 — Figure S5 [file 41420_2023_1400_MOESM7_ESM.pdf]

p value = $4.423e^{-12}$
FDR = $5.519e^{-9}$

1. VEH Wild Type
2. AEA Wild Type
3. VEH $CTNNB1^{CRISPR}$
4. AEA $CTNNB1^{CRISPR}$

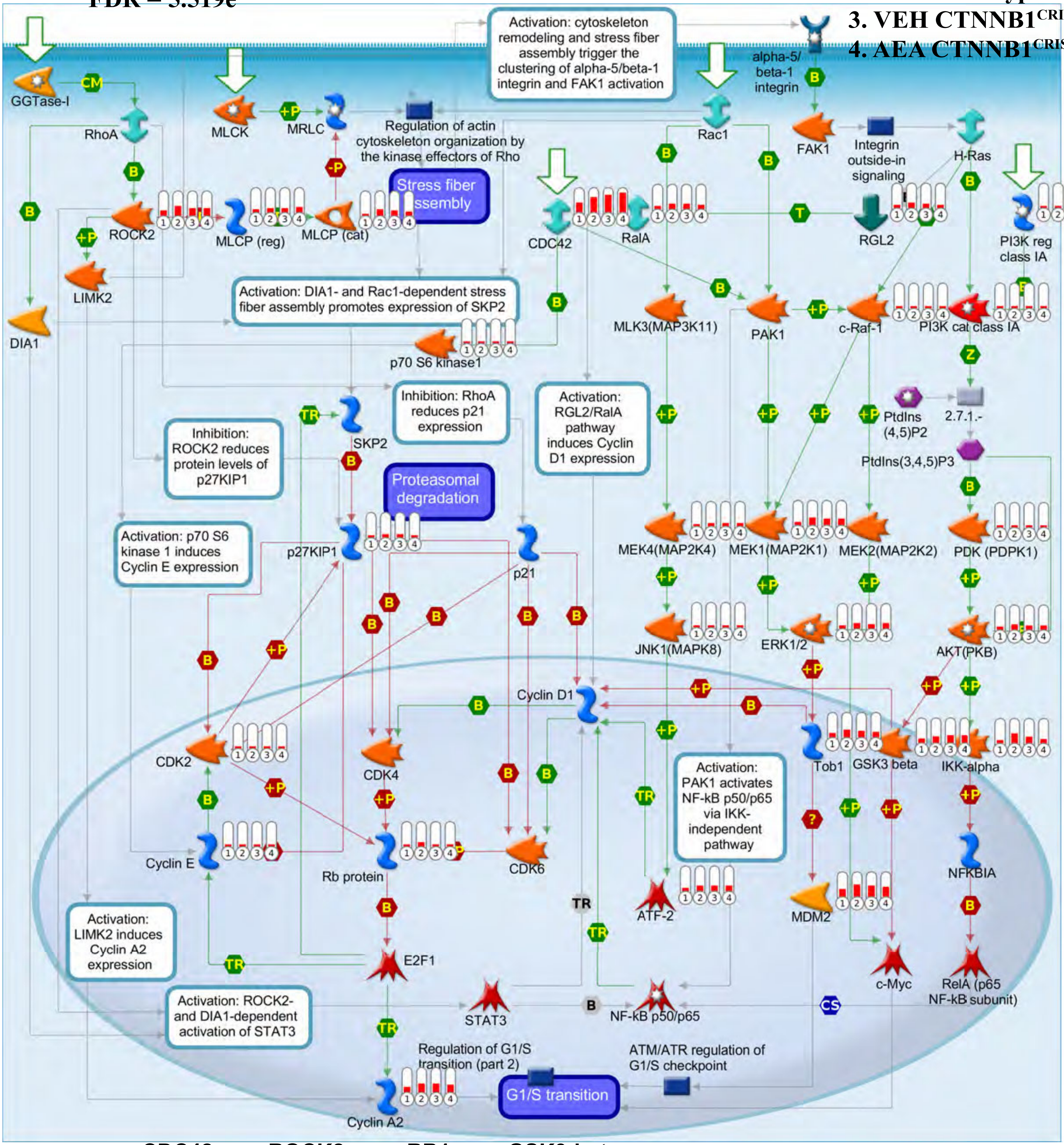

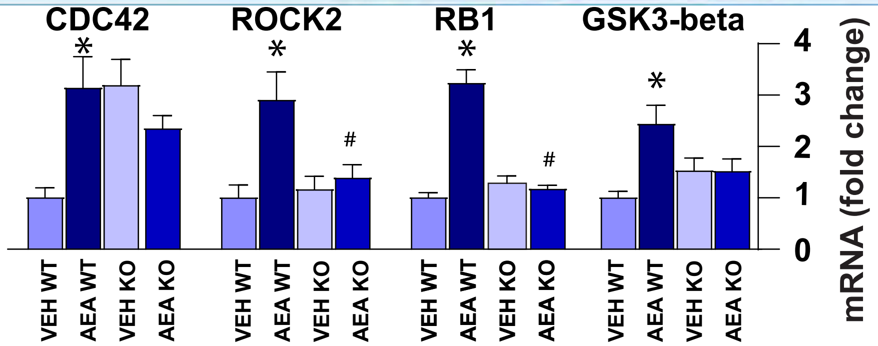

Figure S5
